# Analysis of the Significance of miR-141 and CD147 Expression in Bladder Cancer Cells and Its Relationship with Tumor Grade

**DOI:** 10.1155/2022/1335441

**Published:** 2022-05-17

**Authors:** Chao Liu, Zhijia Li, Lidong Ni

**Affiliations:** Department of Urology, Wenzhou Central Hospital, Wenzhou 325000, China

## Abstract

The study analyzes the significance of miR-141 and extracellular matrix metalloproteinase-induced molecule (CD147) in bladder cancer cells and their correlation with tumor grade. A total of 87 bladder cancer patients for diagnosis and treatment from August 2020 to August 2021 are selected. All patients underwent pathological biopsy, and their cancer tissues and adjacent tissue cells are extracted. RT-PCR is used to complete the miR-141 relative expression level, and the immunohistochemical method is used to detect the expression of CD147 in patients. The miR-141 and CD147-positive rates are compared, and the relative expression levels of miR-141 and CD147-positive rates in cells of different tumor grades are compared. Spearman correlation coefficient is used to analyze the correlation of CD147 and bladder cancer. The prognosis of patients with different expression levels of miR-141 and CD147 is compared. Both miR-141 and CD147 are highly expressed in bladder cancer tissues, and their expression levels are significantly different from those of normal adjacent cells. In addition, the above two indicators are closely related to and related to the tumor grade of bladder cancer patients, and the high expression of miR-141 and CD147 brings the poor prognosis effect to bladder cancer patients.

## 1. Introduction

Bladder cancer is a relatively common disease in clinical urinary system malignant tumor disease, most of which occurs in men. Existing studies have shown that with the change of people's lifestyle, the number of bladder cancer patients is increasing year by year and tends to develop in a younger direction, seriously affecting human life safety and quality of life [1, 2]. Research data show that the recent prognosis in patients with bladder cancer is poorer, case fatality rate is higher, penetrates the disease mechanism of bladder cancer, and clear specific useful tumor marker is currently one of the main topics of the clinical research; its research is the core in order to achieve the early diagnosis, early diagnosis, and intervention for patients with bladder cancer, improve the patient's quality of life, and reduce clinical mortality [3, 4]. The pathogenesis of poor prognosis of bladder cancer patients shows that metastasis and invasion of bladder malignant tumors are important causes of death of patients, so effective control of bladder cancer cell migration and invasion is the key to prolong the life of patients [5]. The extracellular metalloproteinase inducer (EMMPRIN/CD147) has been found to be a unidirectional transmembrane glycoprotein on the surface of tumor cells. Through established experiments, it is found that there is a close relationship between tumor migration and invasion [6, 7]. MicroRNA-141 (miR-141) is an endogenous noncoding RNA that can target to regulate the activities of cancer cells. Studies have shown that this RNA is highly expressed in colorectal cancer [8, 9]. However, there are few studies on the expression of miR-141 and CD147 in bladder cancer tissues and their relationship with tumor grade. Based on this, this study analyzed the expression levels of miR-141 and CD147 in bladder cancer tissues and adjacent normal tissues and explored the relationship between different expressions and tumor grade and patient prognosis, in order to provide basis for clinical treatment of bladder cancer.

A total of 87 patients with bladder cancer admitted from August 2020 to August 2021 are selected. Their cancer tissue cells and normal adjacent tissue cells are collected by pathological biopsy, and bladder cancer cell group and normal adjacent cell group are established, respectively. There are 72 male and 15 female bladder cancer patients, aged from 46 to 70 years old, with an average age of (55.62 ± 4.73) years old. In terms of tumor stages, there are 61 cases of stages I-II, 26 cases of stages III-IV, 41 cases of G1 grade, 46 cases of G2 grade, and 57 cases of patients with signs of tumor metastasis. Inclusion criteria include the following: all patients are diagnosed with bladder cancer by clinicopathological examination; before admission to our hospital for surgical treatment, they did not receive any anticancer treatment; the patients had good clinical compliance and are able to cooperate with the study to complete relevant examinations and follow-up investigations; and all cases are the first time. Exclusion criteria include the following: patients with other malignant tumor diseases; those with a history of mental illness or a manifestation of mental disorder; and poor coordination or withdrawal from the study due to various reasons.

The remainder of this study is organized as follows.  Section 2 presents the detection method.  Section 3 provides its results.  Section 4 analyzes the experimental results. Finally, the conclusions of this study and some future recommendations are given in Section 5.

## 2. The Detection Method

It is performed on the cancer tissues and adjacent tissues extracted from all bladder cancer patients using the real-time polymerase chain reaction (RT-PCR) in this study. Total DNA is extracted using kits provided by Shanghai Yindole Biotechnology Co., Ltd. Reverse transcription of cDNA is achieved using the reverse transcription kit provided by Shanghai Shuhua Biotechnology Co., Ltd. PCR amplification is completed on this basis, and the PCR amplification system is established: CDNA 2.0 *μ*L, primer 1.0 *μ*L, RNase H_2_O 9.5 *μ*L, SYBR Premix Ex TaqTM 12.5 *μ*L, and total reaction system 25 *μ*L. Environmental conditions of PCR reaction are set as follows: the PCR reaction is premodified at 95°C for 5 min, denaturated at 93°C for 10 s, and denaturated at 61°C for 30 s. The reaction mode is cycled for 40 times. It is subjected to 1.5% agarose gel electrophoresis to complete systematic scanning analysis. The amplification curve is generated, and the cycle number (Ct) is read. On the basis of *β*-actin as internal reference, the expression level of miR-141 is analyzed by the relative quantitative method using 2^−△△Ct^. The relative expression level of bladder cancer tissues/adjacent normal tissues ≥6 is determined as high expression of mir-141; otherwise, it is determined as low expression of miR-141 [10].

The samples are equipped with 3% hyaluronic acid (HA) and kept sealed for 20 min. Then, the samples are ished with distilled water PBS, sealed with goat serum for 1 h, and ished with PBS buffer for 3 times. After that, MaxVision reagent provided by Shanghai Bialy Biotechnology Co., Ltd., is added and then placed at room temperature; the samples to be tested are treated by paraffin embedding. After the samples are combined with paraffin and confirmed to be solidified, the samples to be tested are cut by a paraffin slicer, with a thickness of 4 *μ*m chips. The tissue chip is placed in a 65°C constant temperature environment for 5 h before testing.

After discharge, we followed up for 6 months by online and in-hospital revisit to 87 patients and were followed up every 3 months. All the data involved in this study are processed by SPSS 26.0 statistical software, and the relative expression levels of mir-141 mRNA and mir-141 in different cancer cells are compared by the *t-*test, and the relationship between the expression levels of the above two indicators in different stages of bladder cancer is compared. Kaplan–Meier survival curve is used to reflect the survival of patients with different expression levels of mir-141 and CD147. *P* < 0.05 indicates statistical difference.

## 3. The Experimental Results

### 3.1. The Relative Expression Level of miR-141 and Positive Expression Rate of CD147

The expression level of miR-141 and CD147 in the bladder cancer tissue group increased significantly (all *P* < 0.05), as given in Table 1. Table 1 provides the relative expression level of miR-141 and positive expression rate of CD147.

### 3.2. The Relative Expression Levels of miR-141 and Positive Expression Rates of CD147 in Cell Samples of Different Tumor Grades Are Compared

According to the tumor grade of patients with cancer, subgroups are established for the bladder cancer tissue cell group, which are the *G*_1_ grade group (*n* = 41) and G2 grade group (*n* = 46), respectively. It compared the relative expression level of miR-141 and positive expression rate of CD147 and increased with the increase of tumor grade (both *P* < 0.05), as given in Table 2. Table 2 provides the relative expression levels of miR-141 and positive expression rates of CD147 in cell samples of different tumor grades.

### 3.3. Spearman Correlation Coefficient Analysis of the Relative Expression of miR-141 and CD147 and the Tumor Grade of Bladder Cancer

Spearman correlation coefficient analysis showed that the relative expression level of miR-141 and the positive expression of CD147 are significantly positively correlated with the tumor grade of bladder cancer (all *P* < 0.05), as given in Table 3. Table 3 provides the spearman correlation coefficient analysis of the relative expression of miR-141 and CD147 and the tumor grade of bladder cancer.

### 3.4. Comparison of Prognosis of Patients with Different Relative Expression Levels of miR-141

**Figure 1:**
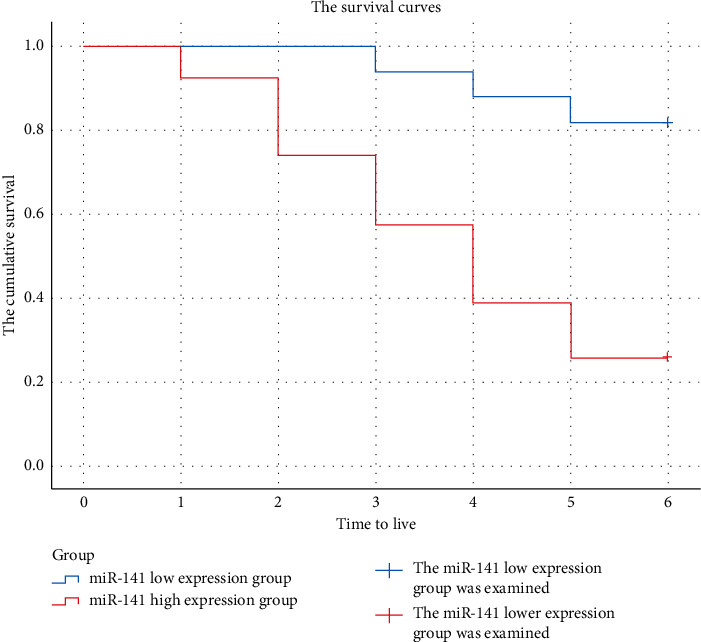
Survival of patients in the two groups after 6-month follow-up.

Subgroups are established according to the relative expression levels of miR-141 in bladder cancer patients. Patients with ≥6 expression levels are the high expression group of miR-141 (*n* = 53) and patients with <6 expression levels are the low expression group of miR-141 (*n* = 34). The overall survival rate and progression-free survival rate of patients with the low miR-141 expression group are significantly higher than those with the high miR-141 expression group (all *P* < 0.05), as given in Table 4, and the survival curve is shown in Figure 1. Table 4 provides the comparison of prognosis of patients with different relative expression levels of miR-141. Figure 1 shows the survival of patients in the two groups after 6-month follow-up.

### 3.5. Compare the Prognosis of Bladder Cancer Patients with Different CD147 Expressions

**Figure 2:**
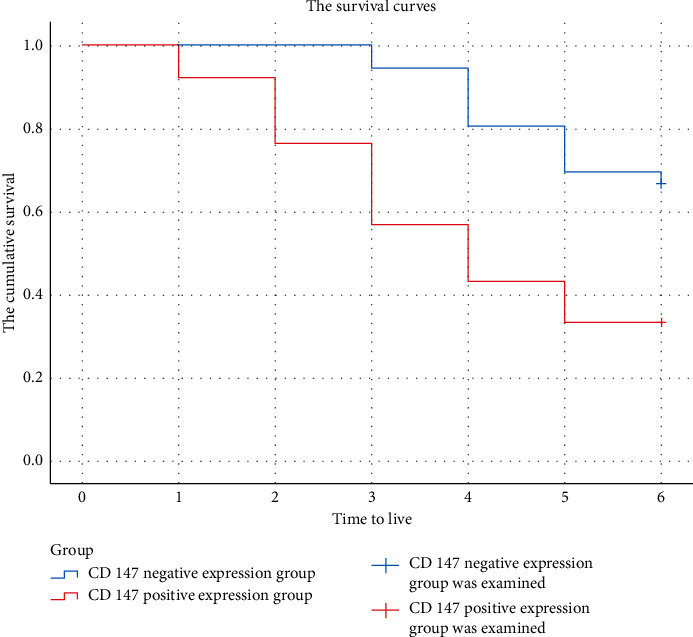
Survival of patients in the two groups after 6-month follow-up.

Subgroups are established according to CD147 expression in bladder cancer patients. Patients with negative expression of CD147 are included in the CD147-negative group (*n* = 36) and patients with positive expression of CD147 are included in the CD147-positive group (*n* = 51). The overall survival rate and progression-free survival rate of patients in the CD147-negative group are significantly higher than those in the CD147-positive group (all *P* < 0.05), as given in Table 5, and the survival curve is shown in Figure 2. Table 5 provides the comparison of prognosis of patients with different CD147 expressions. Figure 2 shows the survival of patients in the two groups after 6-month follow-up.

## 4. The Experimental Result Discussion

Bladder cancer is one of the top ten common tumors with high morbidity and mortality. Clinical analysis of the pathogenesis of bladder cancer shows that about 50% of patients with bladder cancer are caused by smoking, so smoking history is an important factor to increase the risk of bladder cancer. Clinical main symptoms of bladder cancer patients are appearance of blood in the urine, along with the development of the degree of their illness that may appear after urinary frequency, urgency, micturition function decline; some patients with bladder cancer after failing to accept the effective diagnosis and treatment in time, such as progress III B–IV period, after diagnosis have been accompanied by severe regional lymph node metastasis in the distance, and even cause poor prognosis in patients with. Therefore, it is found effective tumor markers for early diagnosis and treatment of bladder cancer.

In this study, the expression of miR-141 and CD147 is compared between bladder cancer tissue cells and normal adjacent cells. It is shown that the relative expression level of miR-141 and positive expression rate of CD147 in cancer tissue cells increased significantly. The expression of miR-141 and CD147 in cancer cell samples is compared again for patients with different tumor grades, and it is shown that the higher the tumor grade, the higher the expression trend of miR-141 and CD147 is, and the expression of miR-141 and CD147 is significantly correlated with the tumor grade. The reason may be related to the action mechanism of miR-141 and CD147 on cells. miR-141 can complement and block the expression of protein-coding genes with targeted regulation of the 3'-end untranslated region and has biological behaviors such as regulating the proliferation and migration of cancer cells, suggesting that its abnormal expression is closely related to the occurrence and development of malignant tumors and can be used as oncogene or tumor suppressor gene to promote or inhibit the occurrence and progression of tumors.

A study on the pathogenesis of colon cancer shows that the polymorphism of miRNAs binding sites is one of the important mechanisms of colon cancer, and the detection of miRNAs expression isolated from feces of subjects has certain effectiveness in early and accurate screening of colon cancer patients to a certain extent. Other studies have reported that miR-141 is highly expressed in the tissues of ovarian cancer patients, and it can be detected in clinical diagnosis of epithelial ovarian cancer to achieve early diagnosis and develop targeted diagnosis and treatment plans. The relative expression level of miR-141 in bladder cancer tissues increased significantly, suggesting that miR-141 is involved in the pathological process of bladder cancer. CD147 is a member of the immunoglobulin family. Clinical experimental results on tumor tissues show that CD147 is often highly expressed in malignant tumor cells, which may be due to the activation of CD147 on fibroblasts and the synthesis of matrix metalloproteinases.

Thus, interstitial components and extracellular basement membrane are degraded, tumor angiogenesis and tumor cell growth are promoted, and the invasiveness of malignant tumor cells in patients is greatly enhanced. CD147 expressed between tumor cells can interact with each other to induce and promote the degradation of the intercellular matrix by matrix metalloproteinases, which contributes to the migration of tumor cells and also increases their invasiveness and metastasis. At present, it has been confirmed that CD147 is highly expressed in a variety of malignant tumors, and the strength of tumor invasion and metastasis can be effectively judged according to its expression intensity.

The results of this study are similar to that. Wong et al. silenced the CD147 gene by small RNA interference, and the bladder cancer cell line activity, proliferation, and migration activity reaction are significantly reduced, and the proliferation, migration, and invasion ability of bladder cancer cell line are significantly decreased, indicating that CD147 is related to the invasion and metastasis of bladder cancer. In addition, follow-up investigation is conducted on patients with different expressions of mir-141 and CD147, respectively, in this study, which showed that the prognosis of patients with low expression of mir-141 and negative expression of CD147 is better, and the overall survival rate is significantly increased (all *P* < 0.05), confirming that the high expression of miR-141 and CD147 brought poor prognosis to patients with bladder cancer.

## 5. Conclusion

The results of this study also have some shortcomings. First of all, a small sample size and short follow-up work may bias the results of this study. Further research should be conducted on the influencing mechanism of prognosis of bladder cancer patients to expand the sample size and extend the follow-up investigation time, so as to further improve the accuracy of the results of this study.

In conclusion, the high expression of miR-141 and CD147-positive in bladder cancer tissues has a direct impact on the activity of bladder cancer cells, will interfere with the occurrence and development of bladder cancer patients, and have a close impact on tumor staging. The above two indicators also have a certain impact on the prognosis and survival rate of bladder cancer patients. Therefore, the above two indicators can be used as the starting point to formulate and improve the clinical diagnosis and treatment plan in the follow-up clinical practice, which also provides new ideas and methods for the control and treatment of bladder cancer metastasis and invasion.

## Figures and Tables

**Table 1:** The relative expression level of miR-141 and positive expression rate of CD147.

Group	miR-141	CD147	
		Negative	Positive
Bladder cancer tissue cell group (*n* = 87)	4.62 ± 0.76	28 (32.18)	59 (67.82)
Paracancer normal tissue cells group (*n* = 87)	0.88 ± 0.12	66 (75.86)	21 (24.14)
*t/x* ^2^	45.339	40.538	
*P*	<0.001	<0.001	

**Table 2:** The relative expression levels of miR-141 and positive expression rates of CD147 in cell samples of different tumor grades.

Group	miR-141	CD147	
		Negative	Positive
*G*_1_ stage group (*n* = 41)	3.74 ± 0.45	21 (51.22)	20 (48.78)
*G*_2_ stage group (*n* = 46)	5.35 ± 0.78	7 (15.22)	39 (84.78)
*t/x* ^2^	−11.603	12.874	
*P*	<0.001	<0.001	

**Table 3:** Spearman correlation coefficient analysis of the relative expression of miR-141 and CD147 and the tumor grade of bladder cancer.

Indicators	Bladder cancer tumor grade	
	*RS*	*P*
miR-141	0.835	<0.001
CD147	0.764	<0.001

**Table 4:** Comparison of prognosis of patients with different relative expression levels of miR-141 (*n*, %).

Group	Overall survival	Progression-free survival rate
miR-141 high expression group (*n* = 53)	14 (26.42)	11 (20.75)
miR-141 low expression group (*n* = 34)	25 (73.53)	23 (67.65)
*x* ^2^	18.590	19.130
*P*	<0.001	<0.001

**Table 5:** Comparison of prognosis of patients with different CD147 expressions (*n*, %).

Group	Overall survival	Progression-free survival rate
CD147-positive expression group (*n* = 51)	17 (33.33)	13 (25.49)
CD147-negative expression group (*n* = 36)	22 (61.11)	21 (58.33)
*x* ^2^	6.584	9.562
*P*	0.010	0.002

## Data Availability

The data used to support the findings of this study are available from the corresponding author upon request.
